# 

**Published:** 1856-04

**Authors:** 


INCLUDING THE

TRANSACTIONS OF THE EPIDEMIOLOGICAL SOCIETY OF LONDON.

EDITED BY

BENJAMIN W. RICHARDSON, M.D.,

PHYSICIAN TO THE ROYAL INFIRMARY FOR DISEASES OF THE CHEST.

NATIONAL HEALTH IS NATIONAL WEALTH.

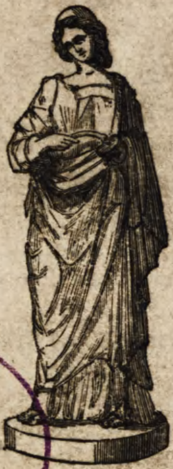

ΥΓΙΕΙΑ

πρεσβίστη μακάρων.

ARMY
MEDICAL
OCT 1 - 1946
LIBRARY

PRINTED AND PUBLISHED FOR THE PROPRIETOR BY

THOMAS RICHARDS, 37, GREAT QUEEN STREET.

1856.

[Published Quarterly.—Annual Subscription, 10s., Payable in Advance.]

